# Ethical triage during the COVID-19 pandemic: a toolkit for neurosurgical resource allocation

**DOI:** 10.1007/s00701-020-04375-w

**Published:** 2020-05-14

**Authors:** Alexander F. C. Hulsbergen, Marleen M. Eijkholt, Naci Balak, Jannick Brennum, Ciarán Bolger, Anna-Margarete Bohrer, Zeev Feldman, Daniel Holsgrove, Neil Kitchen, Tiit I. Mathiesen, Wouter A. Moojen, Nicolás Samprón, Martin Sames, Ulrika Sandvik, Magnus Tisell, Marike L. D. Broekman

**Affiliations:** 1 Ethics Committee of the European Association of Neurosurgical Societies, Brussels, Belgium; 2 Departments of Neurosurgery, Haaglanden Medical Center and Leiden University Medical Center, Lijnbaan 32, 2512 VA The Hague, The Netherlands; 3 Unit Ethics and Health Care, Leiden University Medical Center, Leiden, The Netherlands; 4 Department of Clinical Neuroscience, Beaumont Hospital, Dublin, Ireland; 5 Department of Neurosurgery, Copenhagen University Hospital Rigshospitalet, Blegdamsvej 9, 2100 Copenhagen, Denmark; 6 Department of Clinical Medicine, University of Copenhagen, Copenhagen, Denmark; 7 Department of Clinical Neuroscience, Section for Neurosurgery, Karolinska Intitutet, Stockholm, Sweden; 8 Department of Neurosurgery, Hospital Universitario Donostia, San Sebastián, Spain

**Keywords:** Covid-19, Ethics, Triage, Neurosurgery, SARS-CoV-2

## Abstract

**Background:**

The COVID-19 pandemic confronts healthcare workers, including neurosurgeons, with difficult choices regarding which patients to treat.

**Methods:**

In order to assist ethical triage, this article gives an overview of the main considerations and ethical principles relevant when allocating resources in times of scarcity.

**Results:**

We discuss a framework employing four principles: prioritizing the worst off, maximizing benefits, treating patients equally, and promoting instrumental value. We furthermore discuss the role of age and comorbidity in triage and highlight some principles that may seem intuitive but should not form a basis for triage.

**Conclusions:**

This overview is presented on behalf of the European Association of Neurosurgical Societies and can be used as a toolkit for neurosurgeons faced with ethical dilemmas when triaging patients in times of scarcity.

## Introduction—ethical reflection, why now?

A great shortage of healthcare capacity is looming over us as the COVID-19 (SARS-CoV-2) crisis advances. While at the time of writing, European countries seem to have overcome the initial wave of cases, we are, in the words of German Chancellor Angela Merkel, only at the start of the pandemic [7]. In the future, economic and/or political pressure to alleviate lockdown measures, combined with a risk of faltering public compliance with long-term social distancing requirements, could cause new outbreaks. This creates a realistic possibility of future waves in Europe or elsewhere, which could put healthcare capacity under higher pressure still, either in general or locally.

This may force healthcare workers into painstaking dilemmas. Most COVID-19-related triage befalls general practitioners, internists, and intensivists; however, neurosurgeons, too, will need to triage—first, because insufficient manpower of intensivists may call for other medical specialists to alleviate their burden, and second, because the consequences of scarcity will cascade down into non-COVID patients, including those cared for by neurosurgeons. This “collateral damage”, while typically not incorporated into COVID-19 death tolls, may considerably append the true impact of this pandemic within the neurosurgical patient population.

While the adage “Sometimes wrong, never in doubt” as described by Atul Gawande [4] eloquently describes both a criticism and a strength of surgeons, the choices that need to be made in times of scarcity can be extremely tragic and exert great mental stress on even the most resilient of physicians. Therefore, neurosurgical departments are encouraged to reflect on the ethical dimensions of their triaging policies upfront. While some would argue that this is not the time for ethics—perhaps perceived as long-winded theoretical discourse—the contrary is true: never were the implications of ethical judgments more practical than in times of crisis. Anticipating ethical dilemmas before they happen will save time when they arise and will ease the burden of decision-making “in the field”. Lastly, adhering to prospectively drawn up institutional, regional, or national policies could shield healthcare workers from potential future liability claims.

To help tailor individual triage-related decisions, the Ethico-Legal Committee of the European Association of Neurosurgical Societies presents an overview of ethical considerations relevant for the allocation of medical resources, which can be used as a toolkit by neurosurgeons faced with these dilemmas.

## Selecting patients in scarcity: which principles can we build on?

The traditional pillars of medical ethics (beneficence, non-maleficence, justice, and autonomy) may not provide sufficient guidance in exceptional situations such as a pandemic; they must be complemented by different criteria to guide allocation of scarce resources in a crisis. One possible triage framework has been proposed by Emanuel et al. (NEJM, 2020) [3]. Here, the authors promote allocation according to four values: (1) giving priority to the worst off, (2) maximizing benefits yielded by scarce resources, (3) treating people equally, and (4) promoting and rewarding instrumental value. These four points can be applied in different situations and have different advantages and disadvantages; an overview is presented in Table 1. This framework is not necessarily all-encompassing. For example, to maximize benefits, one still needs to decide whether this constitutes maximizing the number of lives saved versus the number of life-years saved. Below, the four points will be discussed individually.

**Table 1:** Simple principles and their ethical values for resource allocation

**Application**	**Advantages**	**Disadvantages**	**Examples in neurosurgery**	**COVID-application**
**Maximize total benefits**				
Number of lives saved	Saves more lives; benefits the greatest number	Ignores other relevant principles such as sickest first or treating people equally; requires medical assessment	Two patients with EDH are treated instead of one patient with a complex petroclival meningioma	*****
Life-years (maximize survival prognosis)	Maximizes life-years	Ignores other relevant principles; requires medical assessment		*****
Good quality life years	Maximizes quality of life-years, adds dimensions of quality and quantity	Difficult to apply because of valid prognostic factors; problematic assessment of quality, who decides and what is quality; risks random application of cost-effectiveness	Two patients with early herniation from EDH, treating the person who will return to active and conscious life	^
**Treat people equally**				
Lottery or random selection	Facilitates distribution through easy application, hard to corrupt, little information about recipients needed; free from medical assessment	Ignores relevant principles such as saving the maximum number of lives, sickest first…	Clinical research subjects are randomized to placebo or active drug	^^
First-come, first-served	Reflects daily practice; free from medical assessment; little information on recipients needed; available resources are used according to demand	Favors well-connected patients who are likely to present first; ignores other relevant principles like sickest first, youngest first, maximizing life-years.	Neuro-ICU beds go to those who come first as long as available	X
**Favor the worst off**				
Sickest first	Aids those who are suffering right now; appeals to the “rule of rescue” makes sense in temporary scarcity; proxy for being worst off overall	Surreptitious use of prognosis; ignores needs of those who will become sick in future; might falsely assume temporary scarcity; leads to people receiving interventions only after prognosis deteriorates; ignores principles like life-years saved and cost-efficiency	Patient with large acute SDH in deep coma treated before conscious EDH patient with beginning herniation	^^^
Youngest first	Benefits those who have had the least life and experiences^a^; prudent planners have an interests in living to old age	Undesirable priority to infants over adolescents and young adults; ignores other relevant principles.	Pediatric craniosynostosis patients are operated before adults who need cranioplasty after decompressive craniectomy	^^^
**Promote and reward instrumental value (benefit to others)**				
Prospective- priority to those who are likely to make relevant contributions	Promotes continued work and care by health-care personnel,	Vulnerable to abuse through choice of prioritized occupations or activities; can direct health resources away from health needs. Unfair to disadvantaged and could represent misuse of power	Ventilator will go to neurosurgeon over accountant so he can treat multiple brain tumor patients with COVID	^^^
Retrospective-priority to those who have made relevant contributions	Enables promises for those who have contributed to research and exposed themselves to risk (e.g. COVID-19 research and care of virus victims).	Vulnerable to abuse and exploitation; can direct health resources away from health needs; intrusive process of what constitutes a worthy past- contribution;	Brain tumor patient volunteered as research participant early in COVID pandemic to examine effects on the CNS	^^^

Adopted from Persad et al. [6]; based on the idea of “fair” innings as per Callahan D: Setting Limits [1]

*****Priority principle

^Principle to apply with caution: used only in obvious cases, together with other criteria, otherwise too complicated to apply

^^Principle to apply with caution: used for selecting among patients with similar prognosis

^^^Principle to apply with caution: use with maximizing benefits

^X^Principle should not be used

^a^Based on the idea of “fair innings” as per Callahan D: Setting Limits

### Giving priority to the worst off

A classical first line of triage determines which patients most urgently need care. Questions that can be asked to make this decision include the following:

1. Is the patient’s condition *life-threatening*? Will treatment be (potentially) lifesaving? If so, what is the chance that it will work?
2. If the patient’s condition is not life-threatening, will treatment (potentially) prevent *permanent damage*? If so, what is the chance that this will work?
3. If the treatment will not prevent permanent damage, will it prevent or treat *temporary damage*? If so, how much and what is the chance that treatment will work?
4. Can treatment be reasonably *postponed*? If so, how long? Here, one should also take into consideration the expected future capacity based on the distribution of COVID-19 burden over time.

In the context of ICU admission, the life-saving potential of mechanical ventilation in different COVID-19 patient groups is still uncertain. Some reports have demonstrated a high mortality, especially in the elderly [5]. Departments are advised to frequently keep track of institutional or published data to aid decision-making in this respect.

Another dimension of complexity is added by COVID-19: what if, due to infection, the patient leaves the hospital in a worse state than their initial presentation? In line with the principle of nonmaleficence, neurosurgeons have an obligation to assess the risk of infection and unfavorable clinical course for patients who need to be operated, especially those with risk factors such as advanced age. These risks should be discussed with the patient when obtaining informed consent preoperatively.

### Maximizing benefits

Maximizing benefits can be interpreted as either saving the most lives or the most life-years. Both of these approaches can be justified. In scenarios were a certain number of lives could be saved, say, because there is a certain number of ventilators available, determination of life-years may help to triage between two or more patients who would both need one ventilator.

#### Maximizing gained life-years: the role of age and comorbidity

Both a patient’s age and comorbidities play a role in determining potential treatment benefit. For example, older patients or patients with severe comorbidity may have less long-term gain from treatment, even if it is lifesaving in the short term. In the proposed selection criteria, age and comorbidities play a limited, though real, role in the selection. It should be noted that elderly people or people with comorbidities do not principally have less right to healthcare than their healthy, young counterparts, and their life-years count equally. In other words, one gained life year for an 80-year old is as valuable as one gained life year in a 60-year-old cancer patient or a 30-year-old healthy individual. It is therefore not ethical to categorically refuse medically meaningful treatment or intensive care unit (ICU)/hospital admission for older patients, or patients that are impaired by significant comorbidities, against their will. In practice however, younger and healthier individuals will nearly always have the most life-years to gain, which will benefit their triage.

Caution should be applied when making granular estimations of life expectancy. While certain groups in society may have a lower (healthy) life expectancy and, thus, perhaps fewer life-years to gain, this stratification should not be used for triage. Examples may include patients of racial minorities or those of low socioeconomic status. Here, even if such a triage could theoretically be explained by maximizing benefits, this would hit already disadvantaged patient groups and endanger the principle of justice.

#### What constitutes “significant comorbidity?”

Sometimes, comorbidity will be an obvious consideration in triage. Patients with terminal and/or severely debilitating conditions such as metastatic cancer, amyotrophic lateral sclerosis, Alzheimer’s disease, or advanced Parkinson’s disease may have a relatively limited benefit from further treatments when compared with others in situations of dire shortage. Unfortunately, there is a very large gray area as to what constitutes significant comorbidity. There is a broad scale of chronic conditions, including common degenerative conditions (metabolic syndrome, arthrosis, chronic obstructive pulmonary disease), genetic/developmental diseases (autism, hemophilia, Down’s syndrome), or mental health conditions (depression, bipolar disorder, schizophrenia) that may reduce length or quality of life. Taking into account every co-occurrent condition would lead to a draconian triage that again disproportionally affects the already disadvantaged and threatens the principle of justice. Moreover, it ignores the inherent uncertainty of survival prediction. On the other hand, disregarding comorbidity entirely leads to ineffective triage. This article does not aim to advise on where to draw the line, but advises cautious use of comorbidity in triage, weighing beneficence versus justice.

#### Quality of life after treatment

Beyond number of lives and life-years saved, expected quality of life can be an important consideration when determining the merit of treatment. Prolonging life of very low quality may not be desirable, and therefore, quality of life could play a role in triage. However, it should be noted that trying to make granular estimations of quality of life has some considerable limitations. First, we have no data on long-term quality of life after (severe) COVID-19 pneumonia. Second, disease-specific quality of life has a high interpersonal variability; while perhaps measurable on a public health level and useful for global burden of disease estimations, it will be near impossible to accurately assess for individual patients. Third, even if these assessments were roughly feasible, they would be time and resource consuming. Thus, quality of life should only influence triage when there are large and obvious differences between patients.

#### Length of ICU stay

While individual length of ICU stay is hard to predict, data on the average clinical course of ICU-admitted COVID-19 patients is increasingly becoming available [5]. Moreover, experienced neurosurgeons will be able to make educated estimations of the expected necessity of and length of ICU stay for neurosurgical patients. Length of ICU stay can be a consideration in triage. While an extremely utilitarian approach would be to construct a sort of “expected gain in life-years per day in the ICU” metric, we believe such a tool is overly calculating and is not justified for the same reasons granular estimations of life-years and quality of life are unjust, as explained in the previous paragraphs. However, when there are large differences in expected ICU stay between patients of otherwise similar prognosis, this criterium could play a role in triage, favoring patients who would need a considerably shorter ICU stay to recover.

### Treating people equally

In the setting of scarcity, two systems have been proposed to operationalize this principle. Enabling allocation of resources one could opt for a system that treats patients on a first come first-basis, or on basis of a lottery. However, neither system seems appropriate to serve as a sole triage tool.

To allocate treatment for patients by a ‘first come, first served’ basis seems unwise. Such system would encourage crowding or even violence near hospitals, as well as unfairly benefit those living in the proximity of care centers. Moreover, it may disadvantage those who happen to fall ill further into the epidemic instead of in the beginning, perhaps due to stricter adherence to social distancing recommendations [3]. A lottery system could be considered a system of allocation, but only in limited circumstances. Its blinded mechanism seems to offer certain advantages: it offers patients an equal chance and requires little time or knowledge of patient’s history beforehand. However, when patients have very different prognoses, randomization would not maximize benefits. Thus, lottery can be used, but only for patients with roughly similar prognoses.

We would like to stress that in case of equal expected benefit of care, patients with diseases other than COVID-19 have an equal right to healthcare as do COVID-19 patients. Triage should therefore not be based on COVID-19 vs other diagnoses but be open to all patients who might benefit from scarce resources. One exception may be when not treating a COVID-19 patient would lead to an elevated risk of contamination of more patients that cannot be mitigated by quarantine. If necessary, neurosurgeons should advocate for their patients’ access to care and can use the other criteria in this article determine when such access can be justified.

### Promoting and rewarding instrumental value

A person’s benefits and value to the system is a very controversial selection criterion. Generally, triaging decisions should not be based on a patient’s potential benefit to society. Such “perceived usefulness” can be a highly subjective judgments that physicians are not well-equipped to make. Moreover, societal use as a criterium for treatment priority has undesirable implications that threaten the principle of justice and may again be particularly unfair those with chronic diseases or mental/physical handicaps.

However, exceptions to this principle can be made. There are strong arguments in favor of prioritization for healthcare workers who could help patient care during the pandemic. First, their hastened recovery will benefit future healthcare system capacity and the ability to treat more patients in the short or intermediate-term future. Second, it is arguably unfair to those exposing themselves—perhaps even without adequate protection—to the greatest risk of infection by helping others if they have a low chance of receiving adequate treatment when falling ill; this may also cause absenteeism among healthcare workers [3]. Third, infected healthcare workers may become “serial spreaders” infecting many patients they interact with. This violates the ethical principle of non-maleficence and should absolutely be avoided. Another exception could be made those who have a direct responsibility for long-term care of others, such as those caring for multiple people. While case-to-case assessment is essential in this category, potential impact on the quality of life for those dependent on the patient could be taken into account. Lastly, patients and researchers who contribute to critical COVID-19 research could be prioritized to ensure the continuity of this research.

### No-go allocation criteria

We furthermore want to discuss two selection criteria that might be intuitive but that do not feature in Emmanuel’s list. We are adamant that these should *not* be allocation criteria to use for individual providers. First, triage should not be based on risk-related behavior in patients. Recently, news media have featured stories of people willfully ignoring national COVID-19-related recommendations, or even displaying direct risk-seeking behaviors [2]. Indignation toward this is entirely understandable. However, prioritizing care cannot be based on this sentiment. This is comparable to how the right to healthcare for other conditions, such as cancer and cardiovascular diseases, is not affected by a patient’s previous lifestyle and/or management of modifiable risk factors. Second, we do not deem it ethical under any circumstances to triage patients based on wealth, insurance status, or social status. In a broader sense, this also includes patient’s immediate ability or willingness to pay, their societal role (beyond essential functions), caste, nationality, ethnicity, religion, political conviction, gender, sexual orientation, or other personal characteristics not discussed above.

## Conclusion

COVID-19 may confront healthcare workers with extremely difficult dilemmas, and no single algorithm can provide complete guidance nor fully alleviate the heavy burden of triage. Individual triage, including weighing beneficence against justice, is ultimately the responsibility of the treating physician and should be tailored to a particular situation. The preferred degree of application of different principles will vary across Europe, and may depend on cultural and political climates. This paper can be used as a toolkit to structure different considerations that may come into play during such decision-making. Neurosurgeons should strive to maximize benefits of treatment in terms of good life for most, while also prioritizing justice in treatment allocation and protection of those who hold key instrumental value.
